# Mapping the unmet supportive care needs of cancer patients, survivors, and caregivers: results from a cross-sectional survey

**DOI:** 10.1007/s00520-026-10347-0

**Published:** 2026-01-19

**Authors:** Isabella L. C. Mariani Wigley, Davide Ferraris, Samuela Castellotti, Massimiliano Pastore, Serena Barello

**Affiliations:** 1 FinnBrain Birth Cohort Study, Department of Clinical Medicine, Turku Brain and Mind Center, University of Turku and Turku University Hospital, Turku, Finland; 2 Centre for Population Health Research, Turku University Hospital and University of Turku, Turku, Finland; 3 Lega Italiana per la lotta contro i tumori di Milano, LILT Milano Monza Brianza, Milan, Italy; 4 Department of Developmental and Social Psychology, University of Padova, Padua, Italy; 5 WHYpsy Lab, Department of Brain and Behavioural Sciences, University of Pavia, Pavia, Italy

**Keywords:** Cancer survivorship, Supportive care needs, Informal caregivers, Psychosocial oncology

## Abstract

**Purpose:**

Cancer imposes a complex psychosocial burden that extends beyond patients in treatment to include survivors and informal caregivers. While the importance of addressing unmet supportive care needs is increasingly acknowledged, comparative data across these interconnected groups remain scarce. This study aims to descriptively assess and compare the unmet supportive care needs of cancer patients undergoing active treatment, survivors, and informal caregivers, identifying both shared and group-specific patterns across key psychosocial and practical domains.

**Methods:**

A cross-sectional online survey was completed by 208 individuals (patients, *n* = 62; survivors, *n* = 86; caregivers, *n* = 60) who had accessed services through the Italian League Against Cancer (LILT). Unmet supportive care needs and psychological distress were assessed using validated self-report tools, including measures of depressive symptoms, anxiety, perceived stress, and a visual analog stress thermometer. Data were analyzed descriptively. The overlapping index was used to quantify intergroup differences and similarities.

**Results:**

Among the unmet-need profiles reported by patients in active treatment and cancer survivors, the smallest overlap emerged in the information and healthcare service needs domain (*η* = 0.74). Patients reported the highest levels of unmet needs, particularly in the emotional, informational, and practical domains. Survivors showed overall lower unmet needs compared to patients, although they continued to experience challenges in navigating the healthcare system and in the psychological/emotional sphere. Caregivers displayed unmet-need profiles closely mirroring those of patients, with overlap indices above 0.90 across domains, and their main challenges concerned emotional burden, communication, and coordination within the healthcare system.

**Conclusions:**

This study showed distinct patterns of unmet supportive care needs among patients, survivors, and caregivers, with patients reporting the highest unmet needs—particularly emotional, informational, and practical—and caregivers exhibiting profiles closely overlapping those of patients. Present findings support the implementation of integrated, person- and dyad-centered care throughout the cancer continuum. Longitudinal studies are needed to monitor evolving unmet needs and assess dyad-centered intervention models.

**Supplementary Information:**

The online version contains supplementary material available at 10.1007/s00520-026-10347-0.

## Introduction

Over the past two decades, substantial advances in cancer screening, diagnostics, and treatment have led to significant improvements in survival rates, resulting in a growing population of individuals living with and beyond cancer [1]. As cancer increasingly becomes a chronic condition, attention has shifted from the acute phase of treatment to the broader continuum of survivorship and long-term care. This shift has brought into focus the complex and enduring unmet supportive care needs not only of patients but also of cancer survivors and the informal caregivers who support them [2, 3].

Cancer is not a solitary experience; it reverberates through families, households, and broader social networks, creating a web of psychosocial and practical challenges. These challenges manifest as diverse unmet supportive care needs—emotional, psychological, informational, financial, relational, and existential—that vary according to the phase of the disease and the role occupied in the illness trajectory [4, 5]. While the importance of person-centered and family-inclusive cancer care is increasingly acknowledged, the actual delivery of such care often remains focused on clinical outcomes, under-addressing the broader spectrum of unmet needs that affect quality of life, life meaning, and purpose as well as resilience [6].

Cancer survivors commonly experience persistent fatigue, fear of recurrence, difficulties in social and occupational reintegration, and a disrupted sense of identity—phenomena now recognized within the framework of *post-treatment survivorship burden*, which describes how physical, psychological, and social challenges accumulate and collectively impact survivors’ quality of life [7, 8]. In parallel, informal caregivers—often spouses, adult children, or close friends—frequently report high levels of stress, emotional exhaustion, informational gaps, and a sense of invisibility within healthcare systems [9]. Despite mounting evidence of the impact of caregiver well-being on patient outcomes, their unmet needs are rarely assessed systematically or incorporated into routine oncology care planning [10].

To the best of our knowledge, existing research has predominantly examined patients, survivors, and caregivers as separate populations, with limited attention to the interrelated nature of their unmet supportive care needs. Studies employing a unified methodological approach to enable direct comparisons across these groups are still rare, and quantitative investigations aimed at delineating shared vs. role-specific patterns of unmet needs remain largely underexplored. The present study seeks to address these gaps by examining the unmet supportive care needs of three key groups affected by cancer: individuals undergoing active treatment, long-term survivors, and informal caregivers. Here, patients are defined as individuals in active oncological treatment, survivors as those who have been clinically disease-free, and informal caregivers as non-professional family members or close others providing unpaid care. Through a comparative, descriptive approach, we aim to identify both shared and group-specific domains of unmet need across psychological, informational, practical, and relational dimensions. By highlighting convergences and divergences in these unmet needs, our goal is to inform the development of more inclusive, responsive, and person- and dyad-centered models of cancer care that reflect the complex, relational, and longitudinal nature of the cancer experience.

## Methods

### Population and procedures

Data were collected between February and November 2024 through an anonymous online survey hosted on the Qualtrics platform. Eligibility criteria included the following: (a) age ≥ 18 years, (b) fluency in Italian, and (c) having accessed services or received support from one of the participating provincial sections of the Italian League for the Fight Against Cancer (LILT), specifically the sections of Milano-Monza-Brianza, Biella, Genova, Trento, and Treviso. A purposive sampling strategy was employed to ensure the inclusion of cancer patients undergoing active treatment, cancer survivors, and informal caregivers. Informed consent was obtained electronically prior to participation, in accordance with ethical research standards. A total of 300 individuals completed the survey (56% female). Participants with more than 25% missing data were excluded from analysis. Additionally, individuals who completed at least 85% of the items but did not meet the eligibility criteria were also removed from the final sample. The resulting sample included 208 participants (56% female and 44% male), divided into patients in active treatment (*n* = 62), cancer survivors (*n* = 86), and informal caregivers (*n* = 60). Descriptive statistics for the sample are provided in Table 1. The study was conducted in accordance with the Declaration of Helsinki and received approval from the Independent Ethics Committee of the University of Pavia (Protocol No. 219/24).

**Table 1:** Descriptive statistics of demographics in patient (*n* = 62), survivor (*n* = 86), and caregiver (*n* = 60) groups

Variable		Patient	Survivor	Caregiver
Gender	Male	15 (24%)	10 (12%)	14 (24%)
	Female	46 (74%)	76 (88%)	45 (76%)
	Missing data	1 (1%)	0	0
Civil status	Single	13 (21%)	0	0
	Married	38 (61%)	55 (64%)	50 (85%)
	Separated/divorced	4 (6%)	9 (10%)	2 (3%)
	Widowed	7 (11%)	7 (8%)	2 (3%)
	Missing data	0	15 (17%)	5 (8%)
Educational level	None	1 (2%)	1 (1%)	2 (3%)
	Primary school	4 (6%)	2 (2%)	3 (5%)
	Middle school	8 (13%)	11 (13%)	14 (24%)
	High school	27 (44%)	41 (48%)	25 (42%)
	Bachelor	16 (26%)	25 (29%)	13 (22%)
	Postgraduate	6 (10%)	6 (7%)	2 (3%)
Beneficiary of Law 104/1992 provisions	Yes	22 (35%)	15 (17%)	14 (24%)
	No	40 (65%)	71 (83%)	45 (76%)
Parenting responsibilities for children < 18 years	Yes	20 (32%)	32 (37%)	26 (44%)
	No	42 (68%)	54 (63%)	33 (56%)
Receives caregiver support daily	Yes	26 (42%)	19 (22%)	-
	No	36 (58%)	67 (78%)	-
Caregiver-care receiver relationship	Friend	1(3%)	1(3%)	2 (4%)
	Daughter/son	5 (19%)	5 (19%)	23 (44%)
	Partner	7 (26%)	7 (26%)	2 (4%)
	Professional	0	0	0
	Wife/husband	13 (50%)	13 (50%)	25 (48%)
	Missing data	0	0	7 (12%)

### Measures

#### Socio-demographic variables

Participants provided detailed socio-demographic information, including age, gender (with an option to withhold disclosure), and geographical region of residence. Marital status and parental responsibilities (i.e., presence of dependent children living in the household) were also recorded. Educational attainment was categorized on a scale ranging from no formal education to postgraduate qualifications. Employment status was assessed, with specific attention to whether any reported job loss was attributable to the illness experience. Additionally, participants indicated whether they received benefits under Law 104/1992, a legislative framework in Italy that provides protections and social support for individuals with certified disabilities or serious chronic illnesses, including workplace accommodations and caregiver leave. Descriptive statistics for the sample’s socio-demographic characteristics are reported in Table 1, while the geographic distribution of participants is illustrated in Supplementary Material Figure S1.

#### Clinical variables

Cancer patients and survivors undergoing active treatment were asked to report their oncological diagnosis by selecting from a comprehensive list of tumor types (see Table S1). Participants indicated their current disease status, specifying whether they were in remission and, if applicable, whether the duration of remission was less than 5 years or 5 years or more. Additional items explored whether participants were currently engaged in follow-up visits or enrolled in oncological rehabilitation programs. The presence of comorbidities was recorded (yes/no), along with whether the participant received regular support from a caregiver. If applicable, respondents were asked to specify the nature of the caregiving relationship (e.g., spouse, partner, child, friend, and professional caregiver) and whether the caregiver cohabited with them.

Informal caregivers were asked to provide parallel information regarding the individual they cared for. This included the care recipient’s type of cancer, remission status (yes/no), duration of remission (< 5 years vs. ≥ 5 years), participation in follow-up care or rehabilitation programs, and presence of additional comorbid conditions. In addition, the frequency of caregiving (e.g., daily assistance), relationship to the patient, and cohabitation status were measured.

#### Self-perceived depressive, anxiety, stress, and distress symptomatology

To characterize the psychological profile of the sample and provide a more complete description of participants’ emotional functioning, psychological distress was assessed using two complementary measures. The primary instrument was the Italian version of the Depression, Anxiety, and Stress Scale – 21 Items (DASS-21) [11], a validated self-report questionnaire designed to assess three related domains of negative emotional states: depression, anxiety, and stress. Each subscale comprises seven items rated on a 4-point Likert scale ranging from 0 (“Did not apply to me at all”) to 3 (“Applied to me very much or most of the time”), reflecting the respondent’s experiences over the past week. Subscale scores are calculated by summing responses and multiplying by two, in accordance with standardized scoring guidelines. Resulting scores are interpreted using established cut-offs to classify symptom severity into five levels: normal, mild, moderate, severe, and extremely severe (Figure S2).

To capture an additional dimension of subjective distress, participants also completed the distress thermometer [12], a widely used single-item visual analogue scale ranging from 0 (no distress) to 10 (extreme distress). This tool is designed to assess self-perceived distress broadly defined as an unpleasant experience of an emotional, psychological, social, or spiritual nature [13]. The distress thermometer has demonstrated convergent validity with other clinical measures [14, 15] and has been shown to be acceptable to patients in oncology settings [16]. Based on previous findings [14–16], a “traffic light” scoring system has been recommended to guide clinical responses: scores of 0–4 (green) indicate low distress requiring no intervention, scores of 5–6 (yellow) suggest moderate distress meriting closer monitoring, and scores ≥ 7 (red) signal high distress and the need for further clinical discussion or referral for psychosocial support. Distress thermometer scores are illustrated in Figure S3.

#### The supportive care needs scale

To evaluate the specific unmet supportive care needs of patients, survivors, and caregivers, we adapted the Supportive Care Needs Survey for Partners and Caregivers (SCNS-P&C) [17]. Originally developed to assess perceived unmet needs among informal caregivers of cancer patients, the SCNS-P&C consists of 44 items encompassing four major domains: emotional and psychological support, health system and information needs, work and social support, and practical assistance. Each item is rated on a 6-point Likert scale (0–5), with higher scores indicating greater unmet need (e.g., a score of 4 reflects high unmet need, while 5 denotes “not applicable”). The original version has demonstrated robust psychometric properties, with Cronbach’s alpha values typically exceeding 0.80 across subscales [17].

For the purposes of this study, we adapted the SCNS-P&C for use with cancer patients and survivors, in addition to caregivers. The adapted version was reviewed and discussed with a panel of clinicians specializing in psycho-oncology and quality-of-life research. Based on their expert input, two additional items were incorporated to capture (i.e., “receiving information regarding advance care planning (living will)” and “receiving information regarding caregiver support organizations”). The final instrument consisted of 46 items. Descriptive statistics for each item, stratified by group (patients, survivors, and caregivers), are reported in Table S2.

### Data analysis

Given the exploratory nature of the study and the relatively limited sample size, data were analyzed descriptively rather than inferentially [18]. Descriptive statistics were computed for the entire sample as well as separately for each of the three study groups: patients in active treatment, survivors, and informal caregivers. Univariate analyses were conducted to examine the distribution of responses for each item of the adapted SCNS-P&C within the total sample and across subgroups (Figure S4 and S7). To explore potential relationships between items, a set of Pearson correlation coefficients was calculated among all SCNS-P&C items. Correlation matrices were visually represented to identify patterns of item aggregation (Figure S5 and S8). To further investigate the convergence and divergence in supportive care unmet needs across groups, the overlapping index (i.e., *η*) was calculated for key comparisons (patients vs. survivors; patients vs. caregivers). This index quantifies the degree of similarity in unmet need profiles item scores, allowing for an assessment of both shared and distinct unmet needs between the groups [19]. Overlap indices were computed at both the scale-wide and item-specific levels and are reported in Figure S6 and S9. Finally, mean scores for each SCNS-P&C item were calculated and graphically represented to provide a detailed depiction of the most frequently reported unmet needs in each group. All statistical analyses were conducted using R software, with data visualization and computations performed using the ggplot2 [20] and overlapping [19] packages.

## Results

### Participant characteristics

In addition to the characteristics reported in Table 1 and the clinical variables presented in the Supplementary Materials (Table S1), further descriptive data are provided for patients, survivors, and caregivers. Patients were aged between 31 and 83 years (*M* = 56.68, SD = 11.99). Regarding remission status, 16 patients (27%) were in remission, while 43 (73%) were not; among those in remission, 11 had been so for less than 5 years, and 5 for between 0 and 5 years. Eighteen patients in active treatments also reported the presence of other pathological conditions while 43 did not. Survivors ranged in age from 19 to 77 years (*M* = 57.20, SD = 11.94). Among them, 89 were enrolled in a clinical follow-up program (*N* = 5 no) and 25 are following a rehabilitation program due to cancer disease (*N* = 61 no). Caregivers ranged in age from 19 to 89 years (*M* = 57.75, SD = 15.09). Most caregivers (*N* = 36) lived with the person they assisted, and the majority (*N* = 52) reported providing daily care, while 7 did not. Forty-nine did not have a chronic condition while 10 do reported it. As reported in the Supplementary Materials, Figure S2 presents DASS-21 scores across the three groups. The majority of participants exceeded the clinical cut-off thresholds on the DASS-21 subscales, indicating elevated levels of psychological distress. Figure S3 shows the distribution of stress thermometer scores among survivors, patients, and caregivers, illustrating similarly heightened stress levels across groups.

### Unmet supportive care needs

Univariate analyses were conducted to examine the distribution of responses for each item of the adapted SCNS-P&C across the entire sample and within each participant group. The results of these analyses are presented in S4 and S7. Preliminary bivariate analyses revealed that item scores demonstrated clustering patterns consistent with the factorial structure of the original instrument (Figure S5 and S8). Accordingly, results are organized and presented across the scale’s four conceptual domains: *emotional and psychological support* (PE), *information needs* (IN), *work and social support* (WS), and *practical care needs* (PCN). Group comparisons are first reported between patients and survivors, followed by analyses comparing patients and informal caregivers, to highlight both shared and role-specific patterns of unmet supportive care needs.

### Comparing patients’ and survivors’ unmet supportive care needs

**Fig. 1:**
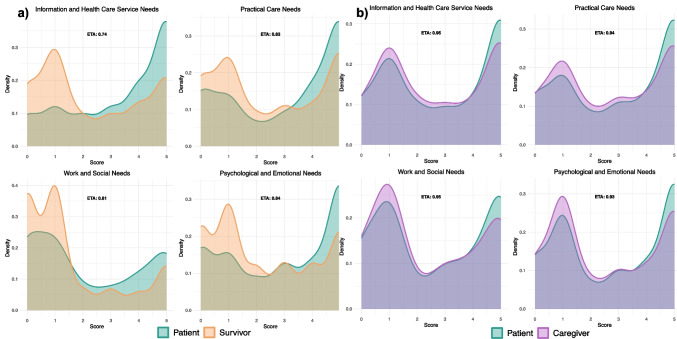
Comparison of density distributions in **a** patients and survivors and **b** patients and caregivers. *η* values are the overlapping indices. The *x*-axis ranges from 0 to 5, where 0 corresponds to “Not appropriate for me”

The overlapping index analysis indicated a high degree of similarity in the overall pattern of unmet supportive care needs reported by patients currently undergoing treatment and cancer survivors across the four core domains of the SCNS-P&C (Fig. 1a). However, notable differences also existed. Specifically, the two groups diverged on the distribution of extreme response values (i.e., scores of 0 and 4), suggesting variability in both the absence of unmet need and in the perception of highly unmet needs. The smallest overlap was observed in the domain of *IN*, with an estimated overlap index of *η* = 0.74.

**Fig. 2:**
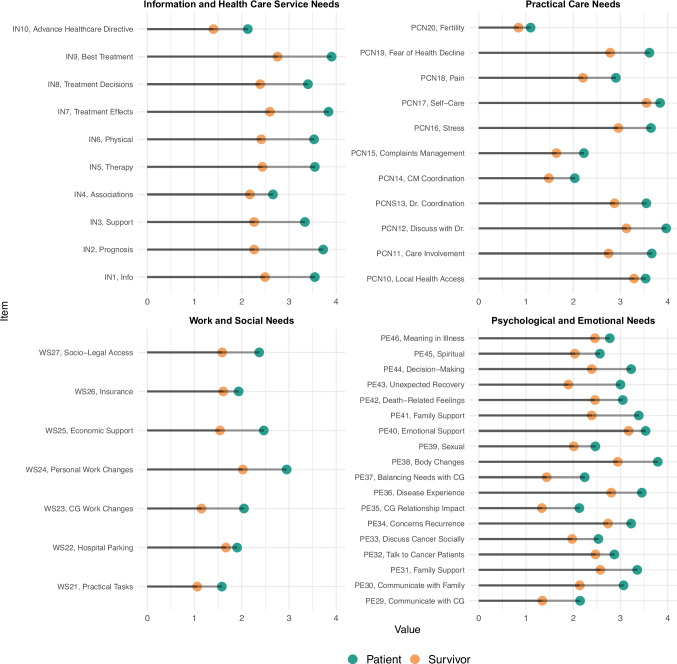
Comparison between patients and survivors regarding unmet work and social needs, psychological and emotional needs, information needs, and healthcare service needs domains. The *x*-axis represents the reported level of need (0–4), and the *y*-axis lists individual items from each domain. In this figure, scores range from 0 to 4 and exclude the “Not appropriate for me” response option

When shifting from domain-level to item-level analyses, results were more differentiated. Patients in active treatment frequently reported higher levels of unmet needs across nearly all items, highlighting their greater burden experienced during this phase of the cancer trajectory (Fig. 2). Within the *IN* domain, patients expressed high levels of unmet needs in most of the areas, with the exception of items IN_4 and IN_10, where no significant difference was observed. In the *PCN* domain, patients reported particularly elevated unmet needs in relation to being actively involved in their care planning (PCN_11), discussing concerns with healthcare professionals (PCN_12), and managing fears related to disease progression (PCN_19). In the *WS* domain, patients endorsed significantly higher levels of unmet need for items related to changes in caregivers’ professional lives (WS_23), access to economic or institutional resources (WS_24), and navigation of social-legal services (WS_25). Finally, in the *PE* domain, the most pronounced differences between patients’ and survivors’ unmet needs were observed in items concerning communication with caregivers (PE_29), communication with family members (PE_30), coping with bodily changes (PE_38), and adjustment to a recovery process that differed from expectations (PE_43). Overlap at the item-specific level is reported in Figure S6.

### Comparing patients’ and caregivers’ unmet supportive care needs

**Fig. 3:**
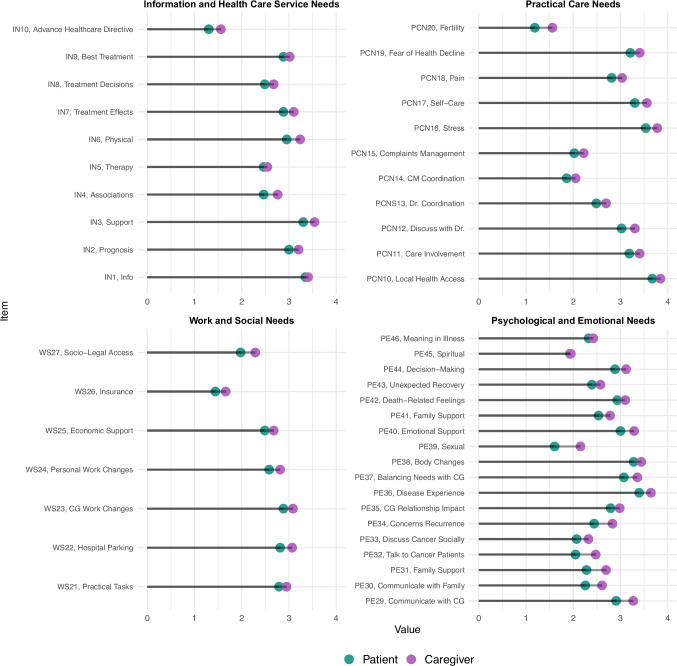
Comparison between patients and caregivers of unmet work and social needs, psychological and emotional needs, information needs, and healthcare service needs. The *x*-axis represents the reported level of need (0–4), and the *y*-axis lists individual items from each domain. In this figure, scores range from 0 to 4 and exclude the “Not appropriate for me” response option

The overlapping index analysis revealed a high degree of convergence between patients and caregivers in terms of perceived unmet supportive care needs (Fig. 1b). Overlap coefficients were consistently high across all four domains, ranging from 0.93 to 0.95, indicating substantial alignment in the type and distribution of reported unmet needs. This overlap was further corroborated by item-level mean score comparisons and graphical representations. While caregivers generally reported slightly higher levels of unmet needs, the magnitude of these differences was minimal. Unlike the distinct patterns observed in the comparison between patients and survivors, patients and caregivers demonstrated remarkably similar mean scores across all major domains. As illustrated in Fig. 3, item-level trends in unmet need intensity closely mirror one another between these two groups, suggesting a shared psychosocial burden and parallel challenges throughout the cancer care trajectory. Overlap at the item-specific level is reported in Figure S9.

## Discussion

This study provides compelling evidence that unmet supportive care needs are not confined to individuals currently undergoing cancer treatment but extend across the broader network of those affected by the disease—namely, survivors and informal caregivers. By adopting a comparative, role-sensitive approach, we delineated both overlapping and distinct patterns of unmet need, emphasizing the heterogeneity of the cancer experience across its various stages and relational contexts. This reinforces a growing body of literature advocating for a systemic, person-centered approach to cancer and to cancer survivorship care [21]. By employing a descriptive and comparative approach, we demonstrated that while certain unmet needs are widely shared, each group also faces distinct challenges that require differentiated responses from health services.

### Principal findings

Patients in active treatment reported the highest levels of unmet need across nearly all domains, including psychological support, healthcare navigation, and practical resources. This aligns with previous research indicating that patients undergoing treatment frequently experience anxiety, depression, and role disruption due to intensive medical interventions and uncertainty about prognosis [22, 23]. Elevated item scores for communication with clinicians, managing treatment side effects, and work-life disruption reflect the emotional and logistical complexity of this phase. Prior studies have shown that clear communication and shared decision-making are central to mitigating psychological distress in oncology settings [24, 25]. Importantly, high unmet needs in emotional domains such as body image concerns, unexpected recovery trajectories, and relational conflict point to the necessity of integrating existential and psychosocial support during active treatment [26].

Survivors, while generally reporting lower levels of unmet need, nonetheless endorsed persistent emotional and relational concerns. Notably, fears of recurrence, social isolation, and challenges adapting to a “new normal” persisted long after the end of treatment. These findings are consistent with the concept of “survivorship distress,” a well-documented phenomenon encompassing anxiety, loss of identity, and uncertainty about the future [27, 28]. The transition from patient to survivor is not merely medical but psychosocial, and often unaccompanied by adequate support systems. Survivorship care plans that incorporate psychosocial screening, peer support programs, and reintegration pathways (i.e., structured processes that support cancer survivors in resuming daily, social, work life after treatment) are increasingly recommended in policy and practice [29]. Moreover, as shown in our comparative analysis, patients undergoing active treatment frequently reported higher levels of unmet needs across all domains when compared to survivors.

These findings underscore that while patients and survivors share many unmet needs, the active treatment phase is characterized by a greater intensity and breadth of unmet needs, particularly within psychosocial and practical domains. Moreover, the persistence of emotional and relational unmet needs among survivors highlights the enduring psychological impact of cancer beyond the cessation of treatment, especially in areas concerning identity redefinition, family role shifts, and existential meaning-making. This supports the need for survivorship care models that integrate long-term psychological and relational support mechanisms.

Perhaps most striking, caregivers reported unmet need profiles that closely mirrored those of patients, and in some domains—particularly those related to healthcare system navigation, emotional distress, and relational strain—the intensity of their unmet needs was relatively more pronounced. These findings are consistent with prior research documenting the “dual burden” shouldered by caregivers [30], who manage both the logistical demands of care coordination and the emotional toll of supporting a loved one, often while neglecting their own well-being [31, 32]. This highlights the importance of moving beyond patient-centric models to embrace inclusive care approaches that recognize and address the unmet needs of informal caregivers as partners in the cancer care journey. Caregivers often report high levels of anxiety, depressive symptoms, and burnout, particularly in the absence of institutional support [32]. Most notably, the findings highlight the significant and often underrecognized unmet needs of informal caregivers. The overlapping index revealed a striking similarity between patients and caregivers across all four domains of unmet supportive needs, with minimal divergence in mean item scores. While caregivers generally reported slightly higher levels of unmet need, the overall pattern mirrored that of the patients, particularly in the emotional, informational, and healthcare system-related domains.

### Clinical implications

These findings reinforce the conceptualization of cancer not merely as an individual medical condition, but as a chronic condition co-experienced by patients and caregivers across the trajectory of illness. The substantial convergence observed between patients and caregivers in both the type and intensity of unmet supportive care needs suggests that caregiving is not an auxiliary or peripheral experience, but a parallel burden deeply intertwined with the patient’s illness journey. Caregivers reported levels of unmet need comparable to—if not slightly higher than—those of patients in active treatment, particularly in domains related to emotional strain, informational burden, and coordination of care. This alignment highlights the inadequacy of traditional care models that focus exclusively on the patient and calls for a more inclusive approach that systematically includes needs assessments and supportive interventions to informal caregivers. Recognizing caregivers as active participants in the care process is not only a matter of ethical and psychosocial responsibility but may also contribute to improved clinical outcomes, patient adherence, and continuity of care [33].

The findings of this study underscore the urgent need to evolve current models of cancer survivorship care toward more inclusive, holistic, and relationship-centered approaches. Several key implications emerge for clinical practice: first, routine assessment of unmet supportive care needs should be standard practice—not only for patients, but also for survivors and caregivers. Integrating structured, multidimensional screening tools into care pathways helps detect unmet needs early and allows for timely, tailored interventions [34]. Second, the significant overlap in patient and caregiver unmet needs underscores the importance of incorporating caregiver support into survivorship programs. Caregivers face emotional and practical burdens and should have access to psychological counseling, navigation services, and targeted education to reduce burnout [35]. Third, interventions should address the patient-caregiver dyad as an interdependent unit. Supporting communication, joint coping, and relational resilience can reduce distress and improve care continuity [36]. Fourth, healthcare professionals must be trained to identify psychosocial needs, communicate empathetically, and connect patients and caregivers with appropriate support—without needing to be mental health experts [37]. Finally, system-level changes are crucial. This includes formal recognition of caregiver roles, integration of supportive services into routine care, and equitable resource distribution. Without structural support, even well-intentioned efforts risk failing. Ultimately, supporting caregivers may improve patient treatment adherence, reduce psychological distress for both parties, and enhance clinical outcomes. Embedding caregiver support in survivorship care fosters a more resilient and coordinated care environment for all involved [38].

### Limitations and future directions

Despite the benefits from assessing multiple unmet needs and psychological factors within a comparative design, this study is not without limitations. The sample size, while adequate for descriptive and exploratory purposes, limits the generalizability of findings. Moreover, due to this limitation, we were not able to conduct a formal validation of the scale; however, we performed a visual inspection of item clustering to assess its coherence. The use of self-reported measures may introduce bias related to social desirability or recall. Moreover, while we adapted a validated instrument to capture unmet needs across groups, further validation of the modified tool is warranted. The cross-sectional design of the present study also represents a limitation that is important to acknowledge.

Future research should expand on these findings through longitudinal designs that capture the evolving trajectory of unmet needs over time, and through the development of dyad-based interventions that address the interconnected experiences of patients and caregivers. Moreover, research efforts should prioritize the inclusion of diverse populations, particularly individuals from underrepresented socioeconomic or cultural backgrounds, whose unmet needs may differ substantially.

## Conclusion

This study demonstrates that unmet supportive care needs extend across the full spectrum of individuals affected by cancer, encompassing not only patients in active treatment but also survivors and informal caregivers. While patients experience the most intense and wide-ranging unmet needs during treatment, survivors continue to face persistent emotional and relational challenges long after therapy ends. Notably, caregivers show unmet need profiles that closely parallel—and in some domains exceed—those of patients, underscoring their critical yet underrecognized role in the cancer care trajectory. Together, these findings highlight cancer as a shared, relational experience and reinforce the necessity of moving beyond patient-centered models toward inclusive, systemic approaches to supportive and survivorship care. Integrating routine needs assessment, caregiver support, and dyadic interventions within clinical pathways will be essential to addressing the complex and interconnected challenges faced by patients, survivors, and caregivers alike.

## Supplementary Information

Below is the link to the electronic supplementary material.

Supplementary file 1 (PDF 1.93 MB)

## Data Availability

No datasets were generated or analysed during the current study.
