# Correction to: Mapping the unmet supportive care needs of cancer patients, survivors, and caregivers: results from a cross-sectional survey

**DOI:** 10.1007/s00520-026-10416-4

**Published:** 2026-02-04

**Authors:** Isabella L. C. Mariani Wigley, Davide Ferraris, Samuela Castellotti, Massimiliano Pastore, Serena Barello

**Affiliations:** 1 FinnBrain Birth Cohort Study, Department of Clinical Medicine, Turku Brain and Mind Center, University of Turku and Turku University Hospital, Turku, Finland; 2 Centre for Population Health Research, Turku University Hospital and University of Turku, Turku, Finland; 3 Lega Italiana Per La Lotta Contro I Tumori Di Milano, LILT Milano Monza Brianza, Milan, Italy; 4 Department of Developmental and Social Psychology, University of Padova, Padua, Italy; 5 WHYpsy Lab, Department of Brain and Behavioural Sciences, University of Pavia, Pavia, Italy

**Correction to: Supportive Care in Cancer (2025)**

10.1007/s00520-026-10347-0

The correct name should be Isabella L. C. Mariani Wigley, not Isabella L. C. Wigley Mariani Wigley.

The publisher acknowledges this mistake and emphasizes that it did not originate from the authors.

The original article has been corrected.

