# Supplementary material for: Mapping the unmet supportive care needs of cancer patients, survivors, and caregivers: results from a cross-sectional survey
Source: Support Care Cancer. 2026 Jan 19;34(2):108. doi: 10.1007/s00520-026-10347-0 (PMC12816088; doi:10.1007/s00520-026-10347-0)
Supplement: Supplementary file 1 — (PDF 1.93 MB) [file 520_2026_10347_MOESM1_ESM.pdf]

Mapping the unmet supportive care needs of cancer patients, survivors, and caregivers: results from a cross-sectional survey

Curated by Isabella L. C. Mariani Wigley & Massimiliano Pastore

Table of contents

1 Introducion 2

2 Demographics and clinical variables 2

3 Overall Univariate Descriptives 5

4 Patient - Survivor 5
4.1 Univariate descriptives in the two groups (patient and survivor) 5
4.2 Bivariate descriptives in Patient and Survivor groups 5
4.3 Overlapping index 5

5 Patient - Caregiver 8
5.1 Univariate descriptives in Patient and Caregiver groups 8
5.2 Bivariate descriptives in Patient and Caregiver groups 8
5.3 Overlapping index 11

1 Introducion

This supplementary material provides detailed descriptive analyses supporting the main findings of our study on unmet supportive care needs among patients currently undergoing treatment, survivors and their caregivers. The supplementary tables and figures aim to offer a comprehensive overview of individual item responses, summary statistics, and subgroup comparisons across relevant demographic and clinical variables. Our descriptive approach highlights patterns in reported unmet needs, frequencies of concern across supportive domains, and variability among different respondent groups. By presenting this additional information, we aim to enhance transparency and allow for deeper insight into the distribution and nuances of unmet supportive needs within the studied population. These materials complement the main text and are intended to assist researchers, clinicians, and policy-makers in understanding the breadth and nature of unmet needs in cancer care from the perspectives of both patients and caregivers.

2 Demographics and clinical variables

The data analyzed in this study were collected via an anonymous online survey conducted between February and November 2024. Recruitment was carried out through selected provincial sections of the Lega Italiana per la Lotta contro i Tumori (LILT), a nationwide nonprofit organization dedicated to cancer prevention and patient support in Italy. Specifically, the sections of Milano-Monza-Brianza, Biella, Genova, Trento, and Treviso collaborated in disseminating the survey among patients in active treatment, survivors and informal caregivers engaged in their services. Participants were eligible if they were 18 years old, fluent in Italian, and had received support through a participating LILT section. The final sample consisted of 208 respondents who met inclusion criteria and had sufficiently complete data for a

The map (Figure S1), visualizes the number of participants from each region. Regions are color-coded according to the count of participants, with the color intensity representing the magnitude (darker colors indicate more participants). The legend on the right quantifies the number of participants per region. Since this is a geographical map, the axes represent longitude (x-axis) and latitude (y-axis), corresponding to the spatial location of each region.

Table S1 shows the distribution of oncological diagnoses across the Patient, Survivor and Caregiver groups. The “Caregiver” group refers to the diagnosis of the cared one, as reported by the caregiver, not their own clinical condition.

Figure S2 shows DASS-21 scores in the three groups. The figure clearly shows that the majority of participants across all three groups exceed the clinical cut-off thresholds on the various DASS-21 subscales, indicating elevated levels of psychological distress. Figure S3 shows the distribution of stress thermometer scores across Patients, Survivors and Caregivers.

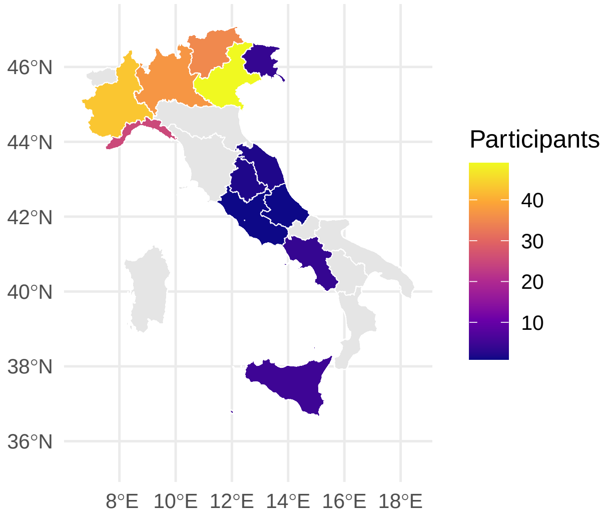

Figure S1: Geographic distribution of participants across Italian regions.

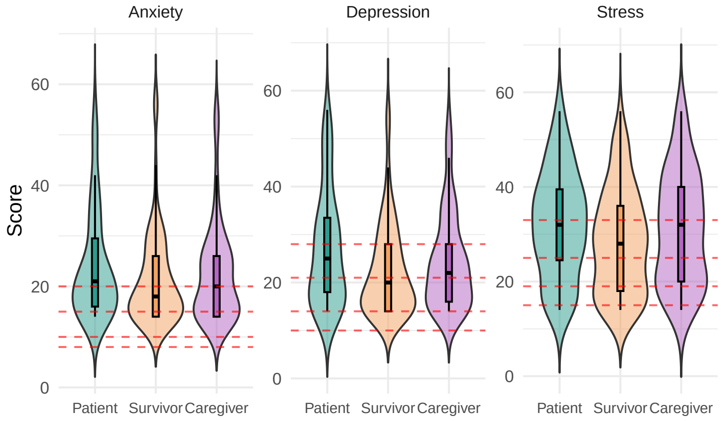

Figure S2: Distribution of DASS-21 scores. Red dashed lines indicate clinical cut-offs for symptom severity levels: mild, moderate, severe, and extremely severe.

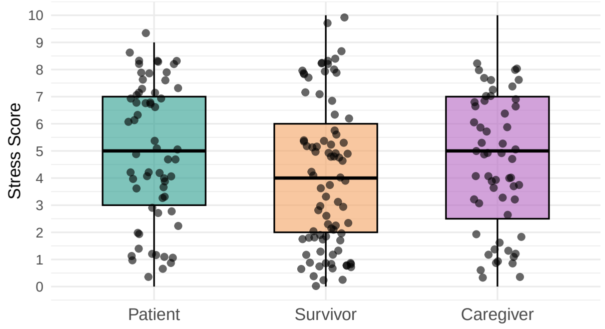

Figure S3: Distribution of Distress Thermometer scores. Each box plot indicates the median and interquartile range. Individual responses are shown as jittered points

Table S1: Oncological Diagnosis in Patient (n = 62), Survivor (n = 86) and Caregiver (n = 60) groups.

**Diagnosis**	**Patient**	**Survivor**	**Caregiver**
Breast cancer	7	58	7
Lung cancer	4	2	2
Prostate cancer	3	2	2
Ovarian cancer	3	3	3
Pancreatic cancer	2	0	0
Stomach cancer	1	0	1
Kidney cancer	1	1	1
Liver cancer	5	0	0
Esophageal cancer	0	0	0
Thyroid cancer	1	0	0
Leukemia	7	4	4
Hodgkin and non-Hodgkin lymphoma	1	1	1
Melanoma	2	1	1
Testicular cancer	1	1	1
Cervical cancer	0	4	4
Sarcoma	1	4	4
Mesothelioma	0	0	0
Head and neck tumors	10	3	3
Renal carcinoma	0	0	0
Ovarian carcinoma	0	1	1
Pancreatic carcinoma	0	0	0
Gastric carcinoma	0	0	0
Hepatic carcinoma	3	0	0
Small cell lung carcinoma	1	0	0
Non-small cell lung carcinoma	0	2	2
Colorectal carcinoma	6	4	4
Prostatic carcinoma	1	1	1
Thyroid carcinoma	0	0	0
Male breast cancer	0	0	0
Adult mesenchymal tumors	0	0	0
Other rare adult tumors	11	5	5

3 Overall Univariate Descriptives

In Table S2 we report descriptive statistics of SCNS-P&C items in the three groups.

4 Patient - Survivor

In this section, we compare unmet supportive care needs between patients currently undergoing cancer treatment and survivors. We begin by presenting the overall univariate descriptive statistics in both groups. Subsequently, we report bivariate statistics within each group to explore associations among SCNS-P&C items. Finally, we include the overlapping index calculated across items, which quantifies the degree of similarity in response patterns between patients and survivors.

4.1 Univariate descriptives in the two groups (patient and survivor)

Figure S4 shows the relative frequencies of key variables across the Patient and Survivor groups. Presenting the data in terms of relative frequencies allows for a clearer comparison of the distributions between these groups, accounting for differences in sample sizes. Each plot is faceted by individual indicators to highlight variations within specific domains.

4.2 Bivariate descriptives in Patient and Survivor groups

Figure S5 displays Pearson correlation matrices of SCNS-P&C items separately for patients and survivors. These visualizations help us explore how the items cluster together and whether their interrelations reflect the original factorial structure proposed by the scale developers. Although a full confirmatory factor analysis would be ideal, the limited sample size makes such an approach impractical due to the large number of parameters that would need to be estimated. Therefore, we rely on bivariate associations as an initial, descriptive assessment of the scale's dimensionality across the two groups.

4.3 Overlapping index

To further explore the comparability of item-level responses between cancer patients and survivors, we examined indices of overlap for each item across the two groups. These overlap measures provide a descriptive quantification of how similarly the items perform in the two populations, independently of specific distributional assumptions.

By visualizing these indices, we aimed to assess the degree to which items are interpreted and endorsed in a consistent manner across patients and survivors (Figure S6).

Table S2: Descriptive statistics of the unmet Supportive Needs Scale in Patient (n = 62), Survivor (n = 86) and Caregiver (n = 60) groups

[!h]

	Patient					Survivor					Caregiver				
item	mean	sd	min	max	median	mean	sd	min	max	median	mean	sd	min	max	median
Q31_1	3.73	1.32	1	5	4.0	2.97	1.58	1	5	3.0	3.53	1.67	1	5	5.0
Q31_2	3.92	1.22	1	5	4.0	2.81	1.72	1	5	2.5	3.63	1.67	1	5	5.0
Q31_3	3.63	1.53	1	5	4.0	2.81	1.68	1	5	3.0	3.73	1.60	1	5	5.0
Q31_4	3.17	1.56	1	5	4.0	2.52	1.52	1	5	2.0	3.40	1.67	1	5	4.0
Q31_5	3.79	1.40	1	5	4.0	2.99	1.72	1	5	3.0	2.94	1.61	1	5	3.0
Q31_6	3.78	1.45	1	5	4.0	2.92	1.68	1	5	3.0	3.41	1.68	1	5	4.0
Q31_7	4.10	1.17	1	5	5.0	3.05	1.70	1	5	3.0	3.27	1.72	1	5	4.0
Q31_8	3.77	1.44	1	5	4.0	2.93	1.67	1	5	3.0	3.04	1.62	1	5	3.0
Q31_9	4.25	1.24	1	5	5.0	3.20	1.68	1	5	4.0	3.24	1.73	1	5	3.0
Q31_10	4.06	1.32	1	5	5.0	3.62	1.56	1	5	4.0	3.91	1.45	1	5	5.0
Q31_11	4.13	1.20	1	5	5.0	3.23	1.67	1	5	3.0	3.59	1.52	1	5	4.0
Q31_12	4.24	1.08	1	5	5.0	3.44	1.58	1	5	4.0	3.48	1.64	1	5	4.0
Q31_13	3.93	1.40	1	5	4.5	3.33	1.59	1	5	4.0	3.06	1.67	1	5	3.0
Q31_14	3.07	1.68	1	5	3.0	2.22	1.46	1	5	1.5	2.81	1.69	1	5	3.0
Q31_15	2.71	1.60	1	5	2.0	2.27	1.52	1	5	1.0	2.85	1.59	1	5	3.0
Q31_16	4.11	1.31	1	5	5.0	3.34	1.59	1	5	4.0	3.98	1.50	1	5	5.0
Q31_17	4.25	1.13	1	5	5.0	3.77	1.49	1	5	4.0	3.75	1.42	1	5	4.0
Q31_18	3.27	1.60	1	5	3.0	2.91	1.72	1	5	2.5	3.38	1.66	1	5	4.0
Q31_19	4.00	1.35	1	5	5.0	3.32	1.60	1	5	4.0	3.87	1.55	1	5	5.0
Q31_20	2.06	1.58	1	5	1.0	1.55	1.25	1	5	1.0	2.97	1.70	1	5	3.0
Q31_21	2.23	1.44	1	5	2.0	1.80	1.33	1	5	1.0	3.35	1.63	1	5	4.0
Q31_22	2.46	1.62	1	5	2.0	2.38	1.74	1	5	1.0	3.48	1.75	1	5	4.0
Q31_23	2.95	1.69	1	5	3.0	2.22	1.62	1	5	1.0	3.64	1.64	1	5	4.5
Q31_24	3.81	1.47	1	5	4.0	2.79	1.69	1	5	3.0	3.39	1.68	1	5	4.0
Q31_25	3.40	1.79	1	5	4.0	2.27	1.60	1	5	1.0	3.10	1.66	1	5	3.0
Q31_26	2.79	1.68	1	5	3.0	2.22	1.63	1	5	1.0	2.23	1.58	1	5	1.0
Q31_27	3.13	1.62	1	5	3.0	2.26	1.64	1	5	1.0	2.76	1.70	1	5	3.0
Q31_28	2.87	1.71	1	5	3.0	2.10	1.52	1	5	1.0	2.49	1.63	1	5	2.0
Q31_29	3.09	1.74	1	5	4.0	2.34	1.66	1	5	1.0	3.64	1.74	1	5	5.0
Q31_30	3.73	1.51	1	5	4.0	2.78	1.71	1	5	2.0	3.14	1.81	1	5	4.0
Q31_31	3.85	1.47	1	5	5.0	3.11	1.74	1	5	3.5	3.18	1.81	1	5	4.0
Q31_32	3.24	1.50	1	5	3.0	2.87	1.52	1	5	3.0	2.75	1.54	1	5	3.0
Q31_33	3.14	1.53	1	5	3.0	2.57	1.44	1	5	2.0	2.58	1.45	1	5	3.0
Q31_34	3.70	1.53	1	5	4.0	3.13	1.46	1	5	3.0	3.55	1.67	1	5	4.0
Q31_35	3.30	1.65	1	5	3.5	2.32	1.61	1	5	1.0	3.38	1.76	1	5	4.0
Q31_36	3.89	1.37	1	5	5.0	3.17	1.53	1	5	3.0	3.84	1.52	1	5	5.0
Q31_37	3.39	1.51	1	5	3.0	2.50	1.59	1	5	2.5	3.60	1.66	1	5	4.0
Q31_38	4.05	1.23	1	5	5.0	3.32	1.57	1	5	4.0	3.76	1.54	1	5	5.0
Q31_39	3.19	1.59	1	5	3.0	2.65	1.63	1	5	2.0	3.10	1.77	1	5	3.0
Q31_40	3.91	1.42	1	5	5.0	3.45	1.54	1	5	4.0	3.46	1.68	1	5	4.0
Q31_41	3.75	1.43	1	5	4.0	2.85	1.63	1	5	3.0	3.22	1.67	1	5	4.0
Q31_42	3.44	1.57	1	5	4.0	2.82	1.60	1	5	3.0	3.52	1.60	1	5	4.0
Q31_43	3.65	1.62	1	5	5.0	2.43	1.58	1	5	2.0	3.23	1.64	1	5	3.0
Q31_44	3.64	1.52	1	5	4.0	2.89	1.56	1	5	3.0	3.29	1.73	1	5	4.0
Q31_45	3.18	1.66	1	5	3.5	2.49	1.48	1	5	2.0	2.56	1.60	1	5	2.0
Q31_46	3.25	1.69	1	5	3.0	2.89	1.66	1	5	2.5	2.98	1.85	1	5	3.5

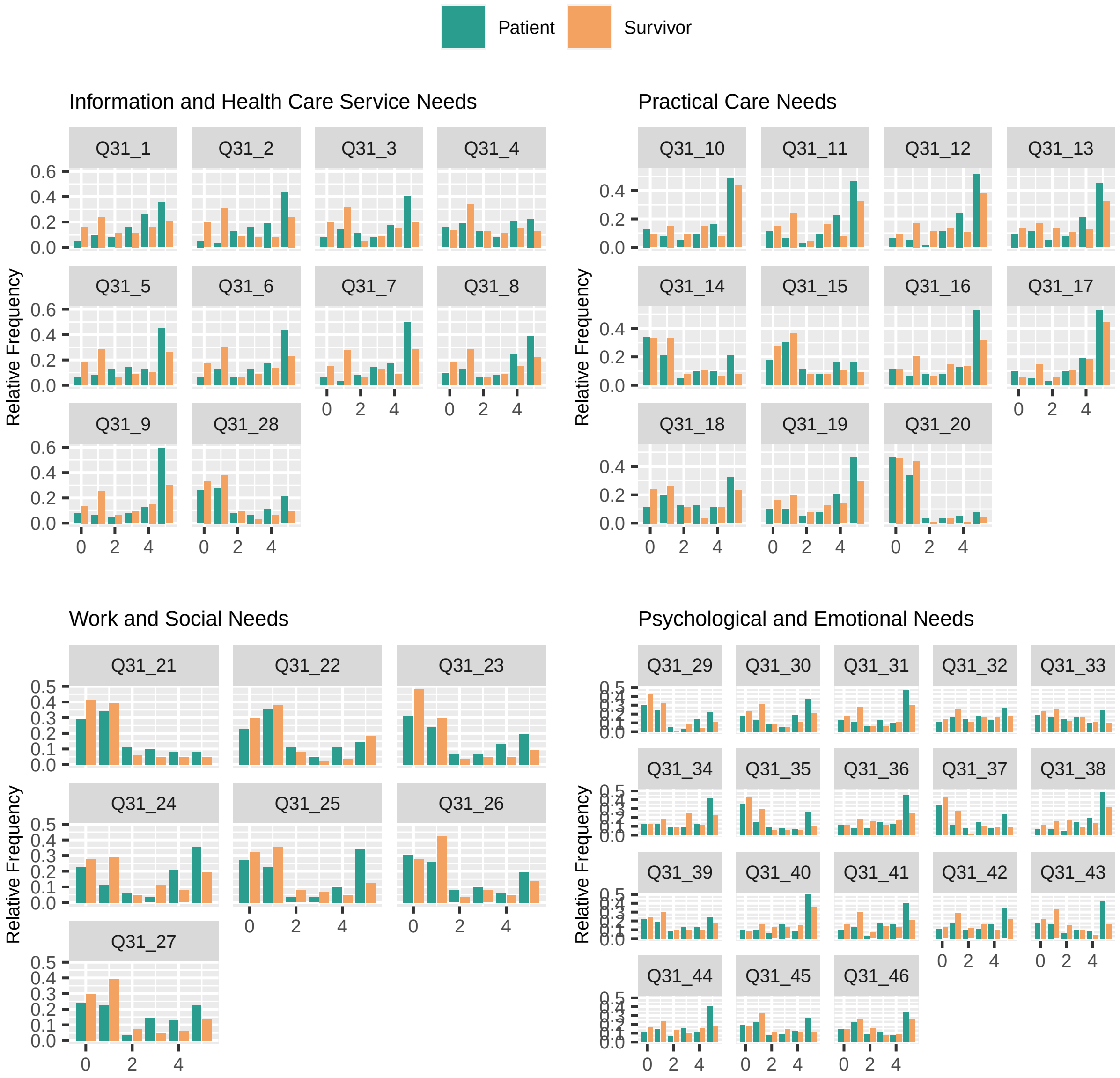

Figure S4: Univariate distribution of SCNS-P&C items in Patient (n = 62) and Survivor (n = 86) groups.

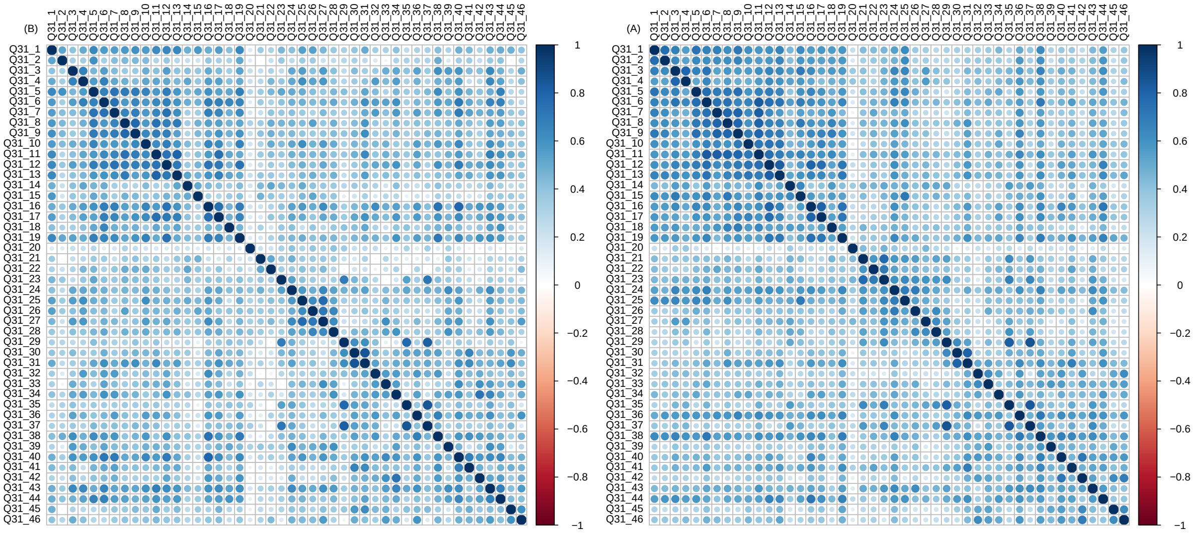

Figure S5: Bivariate distribution of SCNS-P&C items in Patient (n = 62) and Survivor (n = 86) groups.

5 Patient - Caregiver

In this section, we compare supportive care needs between patients currently undergoing cancer treatment and caregivers We begin by presenting the overall univariate descriptive statistics for both groups combined. Subsequently, we report bivariate statistics within each group to explore associations among SCNS-P&C items. Finally, we include the overlap index calculated across items, which quantifies the degree of similarity in response patterns between patients and caregivers.

5.1 Univariate descriptives in Patient and Caregiver groups

Figure S7 displays the relative frequencies of key variables across the Patient and Caregiver groups. Presenting the data in terms of relative frequencies allows for a clearer comparison of the distributions between these groups, accounting for differences in sample sizes. Each plot is faceted by individual indicators to highlight variations within specific domains.

5.2 Bivariate descriptives in Patient and Caregiver groups

Figure S8 shows Pearson correlation matrices of the SCNS-P&C items separately for patients and caregivers. These visualizations help us explore how the items cluster together and whether their interrelations reflect the original factorial structure proposed by the scale developers. Although a full confirmatory factor analysis would be ideal, the limited sample size makes

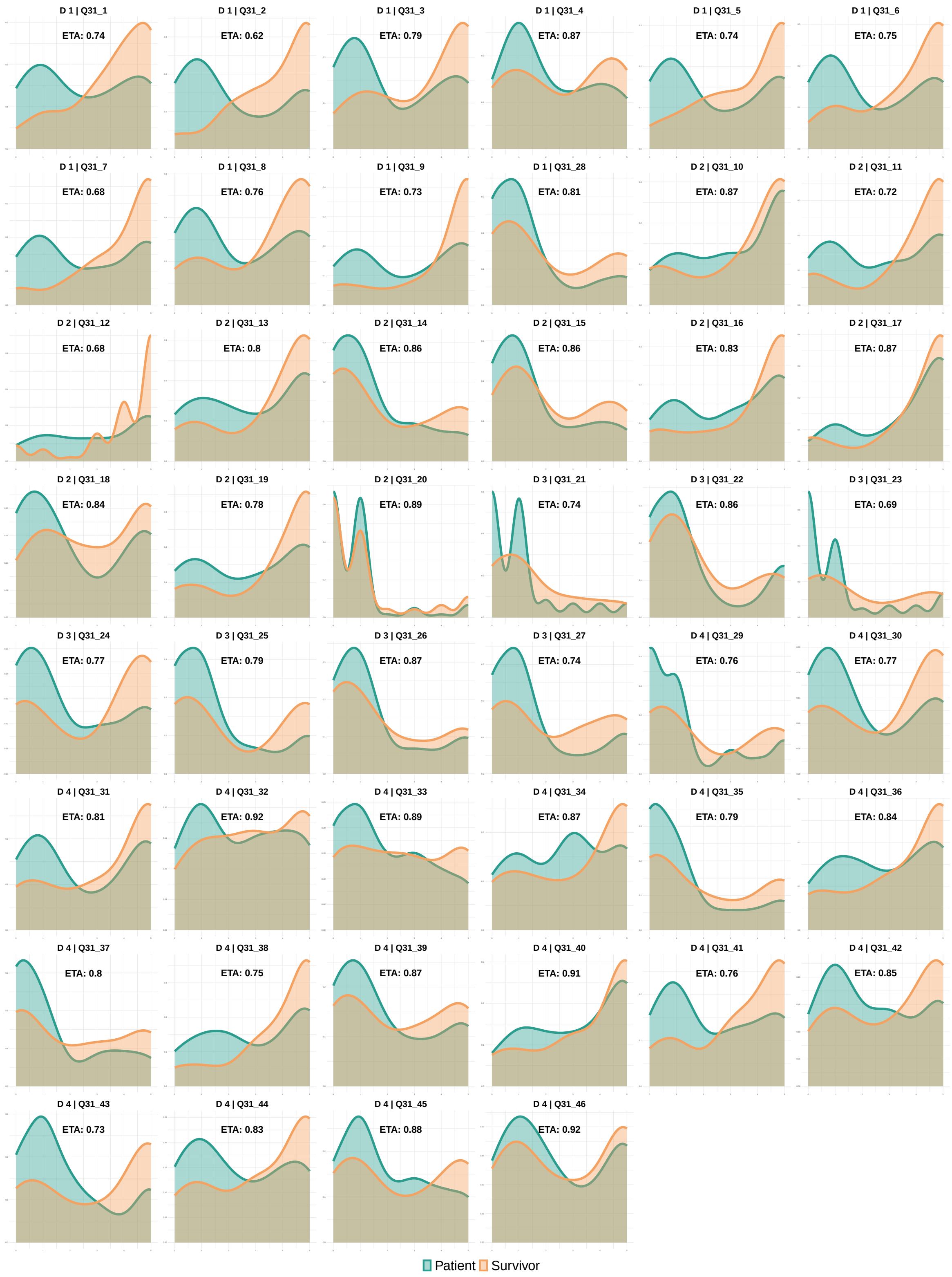

Figure S6: Overlapping index of SCNS-P&C items in Patient (n = 62) and Survivor (n = 86) groups.

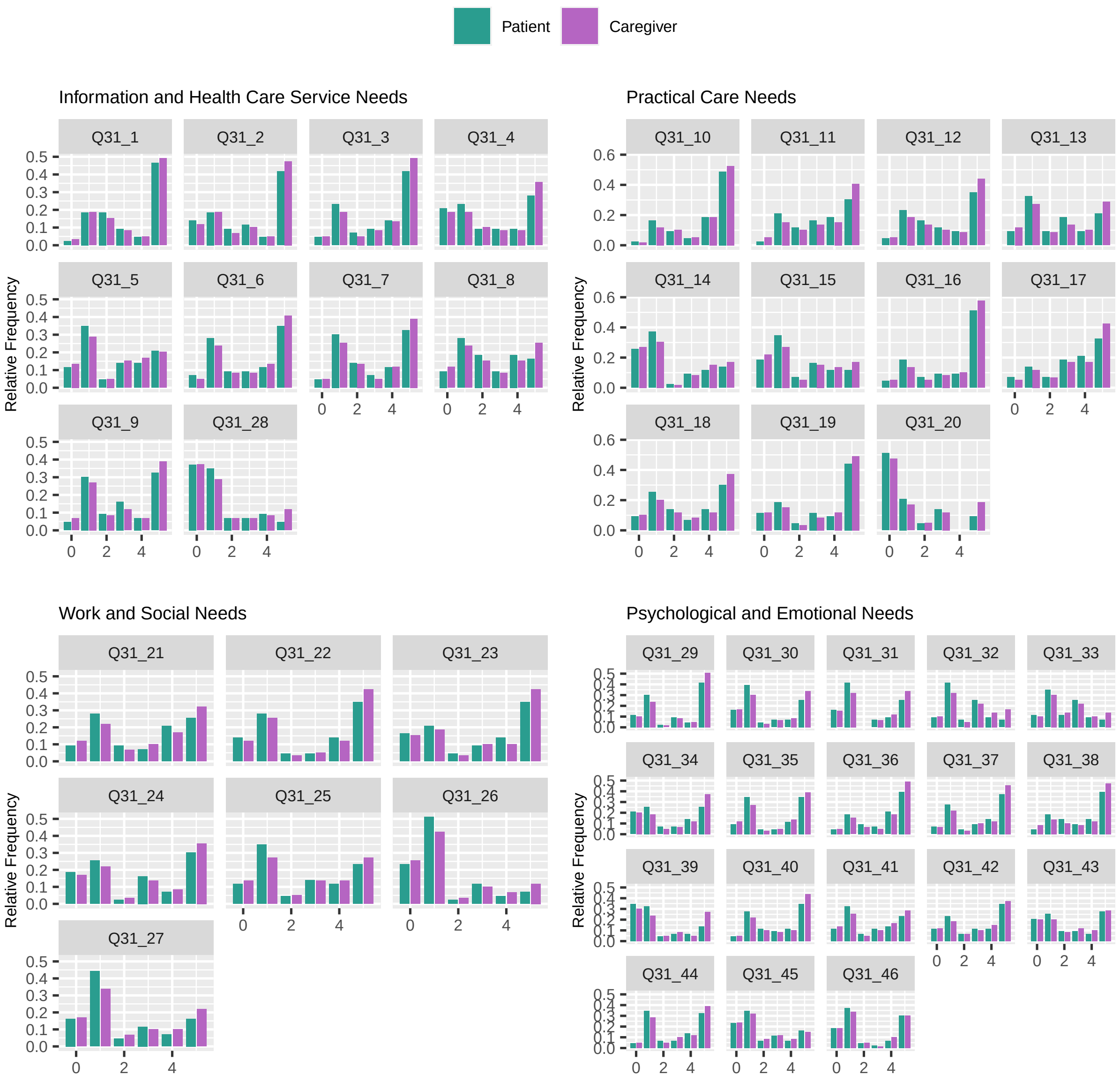

Figure S7: Univariate distribution of SCNS-P&C items in Patients (n = 62) and Caregiver (n = 60) groups.

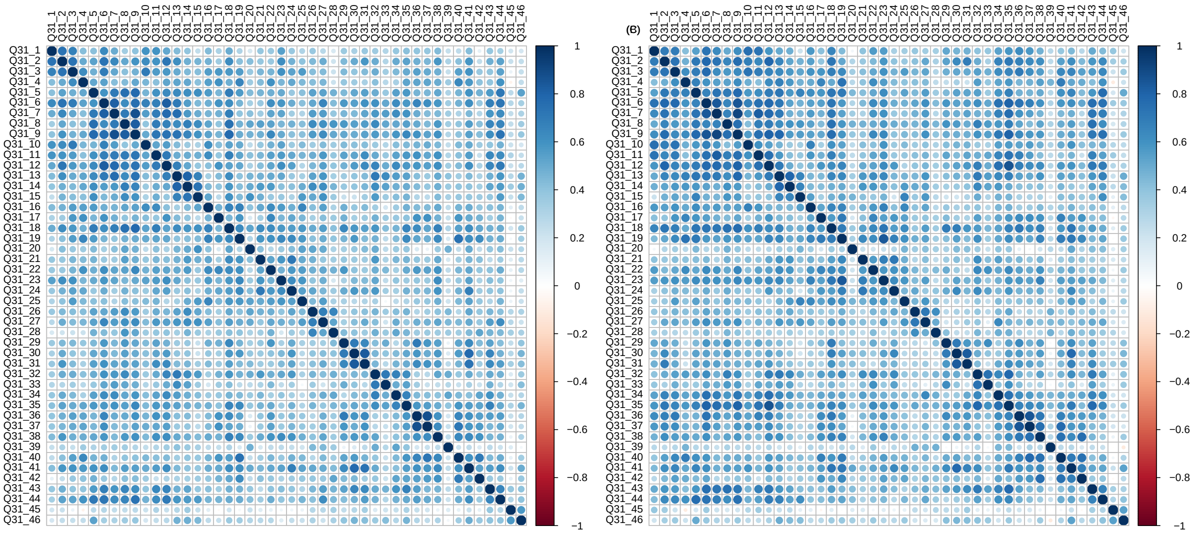

Figure S8: Bivariate distribution of SCNS-P&C items in Patient (n = 62) and Caregiver (n = 60) groups.

such an approach impractical due to the large number of parameters that would need to be estimated. Therefore, we rely on bivariate associations as an initial, descriptive assessment of the scale's dimensionality across the two groups.

5.3 Overlapping index

To further explore the comparability of item-level responses between cancer patients and caregivers, we examined indices of overlap for each item across the two groups. These overlap measures provide a descriptive quantification of how similarly the items perform in the two populations, independently of specific distributional assumptions.

By visualizing these indices, we aimed to assess the degree to which items are interpreted and endorsed in a consistent manner across patients and caregivers (Figure S9).

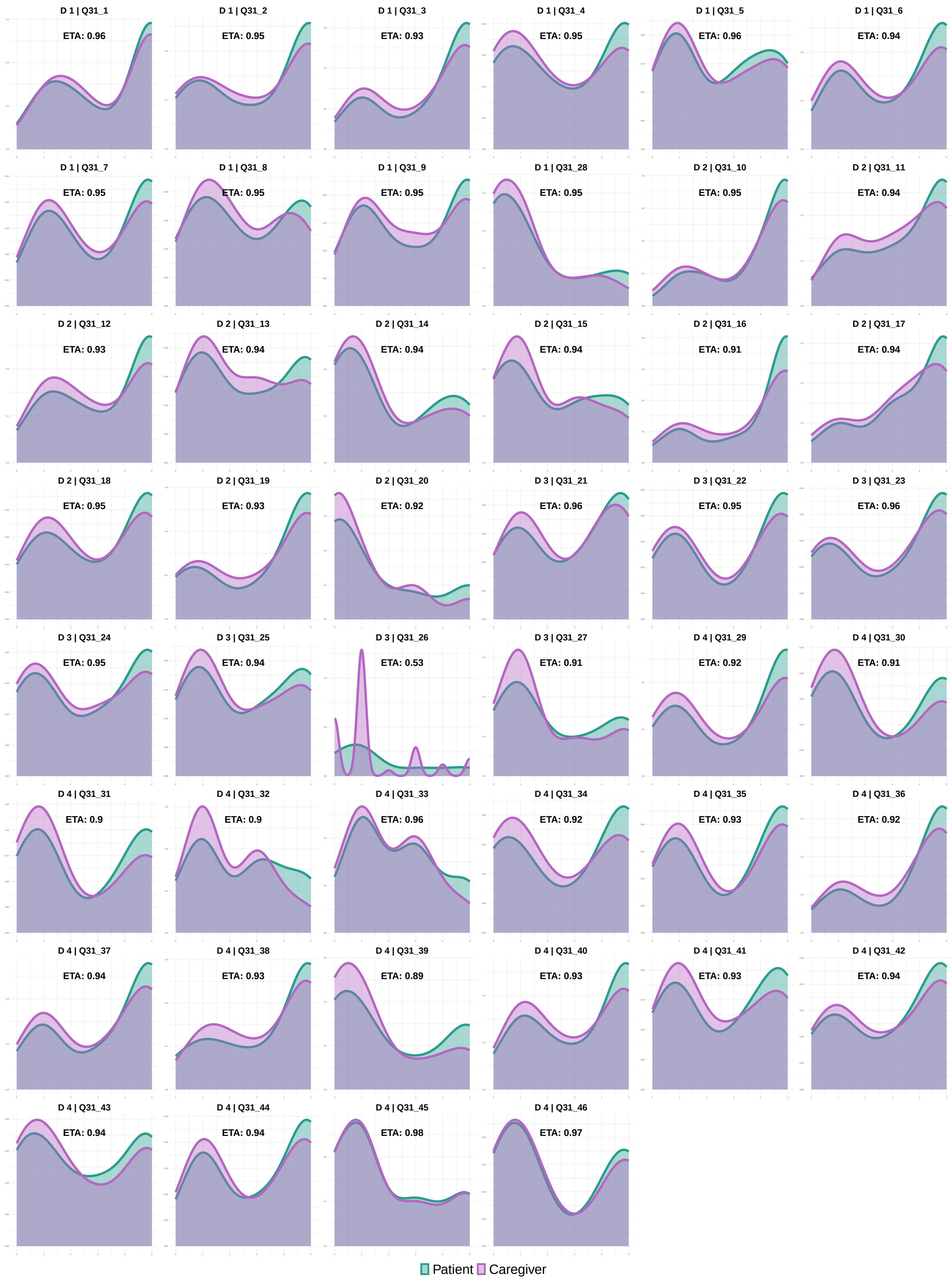

Figure S9: Overlapping index of SCNS-P&C items in Patient (n = 62) and Caregiver (n = 60) groups.
